# Mauriac's syndrome: an uncommon and old complication of type 1 diabetes mellitus

**DOI:** 10.1186/1758-5996-7-S1-A25

**Published:** 2015-11-11

**Authors:** Vânia Balderrama Brondani, Adriana de Mattos Antonio, Mara Barbosa Gayoso, Camila Vicente dos Santos, Rosane Kupfer

**Affiliations:** 1 Institute Estadual de Endocrinologia e Diabetes, Rio de Janeiro, Brazil

## Background

Mauriac's syndrome (MS) may occur in children with poor controlled type 1 diabetes mellitus (DM1) and is associated with growth impairment, puberty delay and hepatomegaly with elevated transaminases. It is a rare syndrome firstly described by Mauriac in 1930 and has become even less common after the emergence of advances on diabetes treatment, but still exists. The exact incidence is uncertain as there are only few cases reported in the literature.

## Objective

This manuscript aims to report the cases of the patients diagnosed with MS hospitalized in Instituto Estadual de Diabetes e Endocrinologia (IEDE), Rio de Janeiro – Brazil, between April/2013 and June/2015, and one patient identified at the outpatient clinic.

## Materials and methods

We performed a retrospective analysis of the records of the patients with diagnostic criteria of MS hospitalized in IEDE between April/2013 and June/2015 and of one patient followed at the outpatient clinic.

## Results

Eight patients with diagnosis of MS were identified and 50% of them were female. The medium age at presentation of MS was 12 (10-14) yrs., with a medium interval between DM1 diagnosis and MS criteria of 6 (3-12) yrs. The medium time of diagnosis of DM1 was 8.12 (5-12) yrs. All the patients were on basal insulin (4 with long-acting insulin analogs and 4 with NPH insulin) and rapid-acting insulin analogs bolus and the medium daily insulin dose was 0.93 (0.61-1.15) U/kg. All had a previous history of poor glycemic control with medium glycated hemoglobin of 11.3 (8.7-14.2%) before the diagnosis of MS. Increase of hepatic enzymes and hepatomegaly at physical examination (4 confirmed by ultrasonography) were present in all the patients. None of the patients had cushingoid features or diabetic microvascular complications. Five patients presented diagnosis of short stature, but growth's deceleration was found in all of them. All the patients presented dyslipidemia. Just 3 patients were adherent to diabetes management and only one of them has never been hospitalized for diabetic ketoacidosis.

## Conclusion

Mauriac's syndrome is an uncommon condition resulting from poorly controlled DM1 in childhood and adolescence. However, staying alert to the existence of MS is fundamental, since most clinical features are reversible with improved glycemic control. Special attention and a multidisciplinary team are necessary to monitor these patients in order to help treatment compliance and prevent complications in the future.

